# A non-invasive method to determine the pluripotent status of stem cells by culture medium microRNA expression detection

**DOI:** 10.1038/srep22380

**Published:** 2016-03-01

**Authors:** Ying Zhang, Gui-Hai Feng, Kai Xu, Libin Wang, Peng Cui, Yuhuan Li, Chenxin Wang, Fei Teng, Jie Hao, Hai-Feng Wan, Yuanqing Tan, Xiu-Jie Wang, Qi Zhou

**Affiliations:** 1 State Key Laboratory of Stem Cell and Reproductive Biology, Institute of Zoology, Chinese Academy of Sciences, Beijing 100101, China; 2 Key Laboratory of Genetic Network Biology, Collaborative Center for Genetics and Development, Institute of Genetics and Developmental Biology, Chinese Academy of Sciences, Beijing 100101, China; 3 University of Chinese Academy of Sciences, Beijing 100049, China; 4 School of Life Sciences, Anhui University, Hefei 230601, China

## Abstract

To precisely determine the type and status of cells is an important prerequisite for basic researches and regenerative medicine involving stem cells or differentiated cells. However, the traditional destructive cell status examination methods have many limitations, mainly due to the heterogeneity of cells under the reprogramming or differentiation/trans-differentiation process. Here we present a new method to non-destructively determine the pluripotent level of embryonic stem cells (ESCs) and induced pluripotent stem cells (iPSCs), or the types of differentiated cells. The method is achieved by examining the expression profiles of microRNAs (miRNAs) in cell culture medium, which show consistent abundance trend as those of the cellular miRNAs. Therefore, the method enables status examination and afterward application being achieved on the same population of cells, which will greatly facilitate cell reprogramming or differentiation/trans-differentiation related based research and clinical therapy.

Cell based therapy is one of the most important aspects of regeneration medicine, for which precise examination of cell status is extremely important before the cells being applied to patients. Both embryonic stem cells and induced pluripotent stem cells (iPSCs) have broad application potentials in regenerative medicine, yet the pluripotent levels of these cells vary a lot among cell lines, batches or colonies. Similarly, the status of differentiated cells, either derived from ESCs/iPSCs or generated via trans-differentiation, is also highly heterogeneous. Therefore, to precisely determine the status of cells is the prior requirement for their basic researches and clinical applications. Yet the current cell status detection methods are mostly destructive, which require to destroy the examined cells. Due to the heterogeneity of cultured stem cells or differentiated cells, such methods therefore cannot guarantee the unexamined cells to have the same status as the examined ones, even when they are in the same culture dishes or colonies. On the other hand, the feasibility of quick determination of cell status in a non-destructive way could offer many advantages. For example, the method could trace the status change of cells along the cell reprogramming or differentiation/trans-differentiation process, therefore to allow fast identification of well reprogrammed or differentiated/trans-differentiated cells, or to compare the effects of different cell reprogramming methods along the reprogramming process. In addition, such non-destructive method will also be of great values for the statue determination of cells with limited resources, such as to evaluate the quality of artificial fertilized embryos.

MicroRNAs (miRNAs) are a class of ~22 nucleotide noncoding RNAs with essential roles in regulating cell fate and functions^1^,^2^,^3^,^4^,^5^. It has been demonstrated that miRNAs collected from various body fluids, such as blood, urine and salivary, can serve as markers for a wide range of diseases or physiological change, including cancers^6^,^7^,^8^,^9^,^10^, diabetes^7^ and tissue injuries^11^,^12^,^13^. During the cell culture process, miRNAs within cells could be released to the culture medium either from the exosomes of cells, or from the damaged cells, therefore could be detected in the culture medium. Here, we report a non-destructive method to determine the type or status of cells by examining the expression profiles of miRNAs in cell culture medium, which will facilitate studies or clinical therapies related to cell reprogramming or differentiation/trans-differentiation.

## Results

### MiRNA expression abundance in mouse cells and cell culture mediums is highly correlated

To examine whether miRNAs collected from cell culture medium can be used to evaluate the status of cells, we first extracted miRNAs from mouse ESCs, iPSCs, embryonic fibroblasts (MEFs), tail tip fibroblasts (TTFs), and their corresponding culture mediums, respectively. A stem-loop reverse transcription PCR (RT-PCR) assay was adapted to examine the expression of mature miRNAs in each sample. Consistent with the previously reported ESC and iPSC specific expression pattern^14^,^15^, high expression of two ES cell cycle regulating (ESCC) miRNAs, miR-292-3p and miR-294, was detected in ESCs and iPSCs, as well as their culture mediums, but were absent in both cells and culture mediums of differentiated MEFs and TTFs (Supplementary Fig. S1A and Fig. S1B). To the contrary, a fibroblast specific miRNA, miR-214, was only detected in the cells and culture mediums of MEFs and TTFs (Supplementary Fig. S1C and Fig. S1D). For all detected miRNAs and cell types, the expression of miRNAs in cells and corresponding cell culture mediums showed the same abundance trend. We also found that the values of miRNAs in culture media were positively correlated with the cell density. To normalize the value in culture medium, we calculated the relative value of the detected miRNA to the reference miRNA U6. The relative values of miRNAs were constant in different cell density (Supplementary Fig. S1E). In order to know whether the ratio of the miRNA amount in culture medium to that in cells was constant, ESCs and iPSCs were cultured under 2i and KOSR culture conditions and MEFs were cultured under FBS and KOSR culture conditions^16^, respectively, then we examined the ratio of the miRNA amount in culture medium to that in cells. We found that the ratios of the miRNA amount in culture medium to that in cells were constant and not changed with cell conditions (Supplementary Fig. S1F). To detect the miRNAs in culture medium, no less than 5000 cells were needed.

### MiRNAs in cells and cell culture mediums exhibited same expression changes during the iPSC generation process

**Figure 1:**
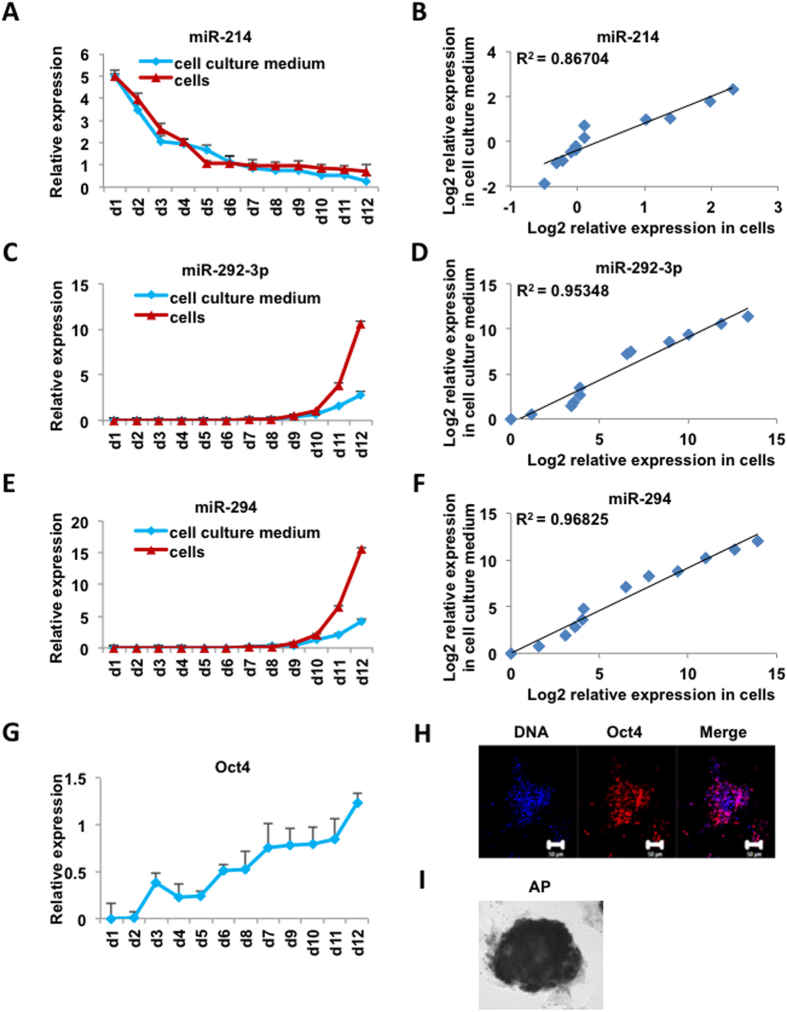
Expression profiles of miRNAs in cells and cell culture mediums during the iPSC generation process. (**A**) Relative expression levels of miRNA-214 in cell culture mediums and cells evaluated by real-time PCR. (**B**) Correlation analysis of miRNA-214 expression (log2 transformed) in cell culture mediums and corresponding cells (p-value = 1.086e-05, two-sided Pearson correlation analysis). (**C**) Relative expression levels of miRNA-292-3p in cell culture mediums and cells evaluated by real-time PCR. (**D**) Correlation analysis of miRNA-292-3p expression (log2 transformed) in cell culture mediums and corresponding cells (p-value = 5.47e-08, two-sided Pearson correlation analysis). (**E**) Relative expression levels of miRNA-294 in cell culture mediums and cells evaluated by real-time PCR. (**F**) Correlation analysis of miRNA-294 in cell culture mediums and corresponding cells (p-value = 8.052e-09, two-sided Pearson correlation analysis). (**G**) Relative expression levels of Oct4 in cells evaluated by real-time PCR. (**H**) Immunostaining of Oct4 protein in iPSCs. Positive Oct4 fluorescence (red) was observed, DNA was stained by Hoechst 33342 (blue). Shown are mouse iPSCs generated in KOSR-based medium. (**I**) AP-positive clones obtained from reprogrammed MEF cells. The x-axis of (**A**), (**C**), (**E**), (**G**) represents the dates during the reprogramming process. Pluripotent iPSCs were obtained at day12.

To further investigate whether the expression of miRNAs in cell culture medium can serve as a marker to evaluate the status of cells, we examined the correlation of miRNA abundance in the cells and cell culture medium during the reprogramming process of MEFs to iPSCs. As expected, the abundance of the fibroblast specific miRNA miR-214 decreased along the reprogramming process in both the cells and cell culture medium (Fig. 1A), whereas the abundance of the iPSC highly expressed miRNAs, miR-292-3p and miR-294, increased gradually (Fig. 1C,E). Statistical analysis revealed that the cellular and culture medium expression of miRNAs were highly correlated (Fig. 1B,D,F). To validate whether the expression change of miRNAs was indeed correlated with the physiological change of cell status, we examined the expression profile of a pluripotency marker gene, Oct4, during the iPS cell generation process. Similar to the profile of pluripotency-related miRNAs, the abundance of Oct4 mRNA gradually increased during the reprogramming process (Fig. 1G). Immunostaining also detected the expression of Oct4 (Fig. 1H) and alkaline phosphatase (AP) (Fig. 1I) in the post-reprogramming cells, indicating that the cells had been converted from MEFs to iPSCs.

### Fully and partially pluripotent stem cells exhibited expression difference of pluripotency related miRNAs in both cells and cell culture mediums

**Figure 2:**
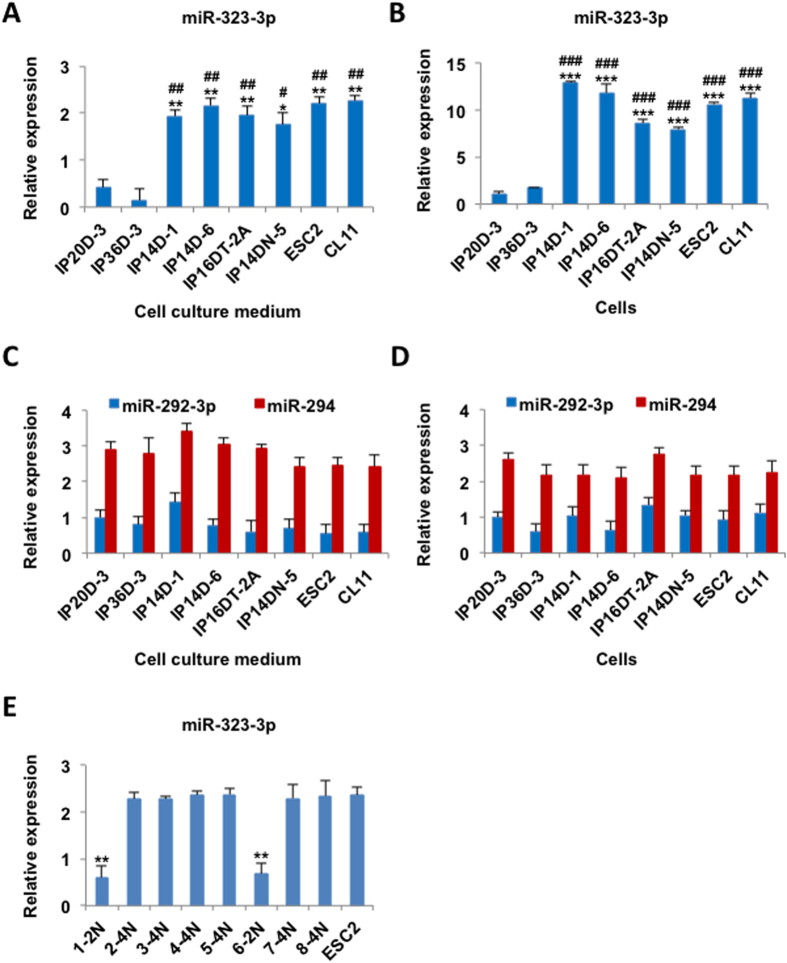
Expression difference of pluripotency related miRNAs in cells and cell culture mediums of fully and partially pluripotent stem cells. (**A**) Relative expression levels of miRNA-323-3p in cell culture mediums of partially pluripotent iPSCs (IP20D-3 and IP36D-3), fully pluripotent iPSCs (IP14D-1, IP14D-6, IP16DT-2A, IP14DN-5) and ESCs (ESC2 and CL11), evaluated by real-time PCR. (**B**) Relative expression levels of miRNA-323-3p in cells evaluated by real-time PCR. (**C**) Relative expression levels of miRNA-292-3p and miRNA-294 in cell culture mediums evaluated by real-time PCR. (**D**) Relative expression levels of miRNA-292-3p and miRNA-294 in cells evaluated by real-time PCR. *P < 0.05, **P < 0.01, ***P < 0.001, compared to IP20D-3, ^#^P < 0.05, ^##^P < 0.01, ^###^P < 0.001, compared to IP36D-3, the Student’s t-test. All the data represent three independent experiments. (**E**) Relative expression levels of miRNA-323-3p in eight iPS clones and ESCs cell culture mediums evaluated by real-time PCR. 2N represents partially pluripotent stem cells and 4N represents fully pluripotent stem cells. **P < 0.01, compared to ESC2, the Student’s t-test. All the data represent three independent experiments.

Previously we have demonstrated that the expression levels of miRNAs within the *Dlk1-Dio3* region of the mouse genome are positively correlated with the pluripotent levels of mouse ESCs or iPSCs^17^. To examine whether the culture medium miRNAs can be used to distinguish ESCs or iPSCs with different pluripotent levels, we detected the expression of an miRNA of the *Dlk1-Dio3* region, miR-323-3p, in different mouse iPSCs with full and partial pluripotency (Table 1). Consistent with previous report^17^, miR-323-3p was highly expressed in cells and cell culture mediums of all examined mouse ESCs and fully pluripotent iPSCs (Fig. 2A,B), but was barely detected in iPSCs with partial pluripotency (IP20D-3 and IP36D-3). Based on the above evidence, the ESC and iPSC highly expressed miR-323-3p could be used as a marker to evaluate the pluripotency level of iPSCs. For other miRNAs with high expression in ESCs but are unrelated to the pluripotency of cells, such as miR-292-3p and miR-294, no expression difference was detected among fully and partially pluripotent cell lines in both cells and cell culture mediums (Fig. 2C,D). These results indicated that the expression of miRNAs in cell culture medium can be used to evaluate the pluripotent level of iPSCs when the right marker was chosen. To demonstrate this method can predict which clones were fully reprogrammed, we cultured eight iPS clones and detected the expression of miR-323-3p in cell culture mediums. We found that the expression of miR-323-3p in two clones were much lower than that in ESCs (ESC2) and the other six clones had the same high miR-323-3p expression level as ESCs. We further detected the pluripotency levels of these iPS clones. We found just these six clones with high miR-323-3p expression with the ability to generate tetraploid complementated embryos (4N) exhibited fully reprogrammed whereas the two clones with low miR-323-3p expression that were only able to produce diploid chimeric mice (2N) were classified as partially pluripotent (Fig. 2E).

### Correlation of miRNA expression with cell status in human cell culture mediums

We next examined whether the expression correlation of cellular and cell culture medium miRNAs is a common feature of cells from different species. To address this question, we detected the expression of marker miRNAs in human ESCs, iPSCs, human fetal fibroblast cells (hFFs) and several cancer cell lines (MCF-7, BEL-7402, A549). Similar to the results in mouse, the ESC and iPSC specific miRNAs, miR-292–3p and miR-294, were highly expressed in human ESCs and iPSCs as well as cell culture mediums, whereas the fibroblast highly-expressed miRNA, miR-214, was abundant in hFFs (Supplementary Fig. S2A, S2B, S2C and S2D). None of these miRNAs were detected in the cells and cell culture mediums of cancer cells, which was consistent with expectation (Supplementary Fig. S2A, S2B, S2C and S2D).

### Cell type determination through miRNA expression detection in cell culture mediums

**Figure 3:**
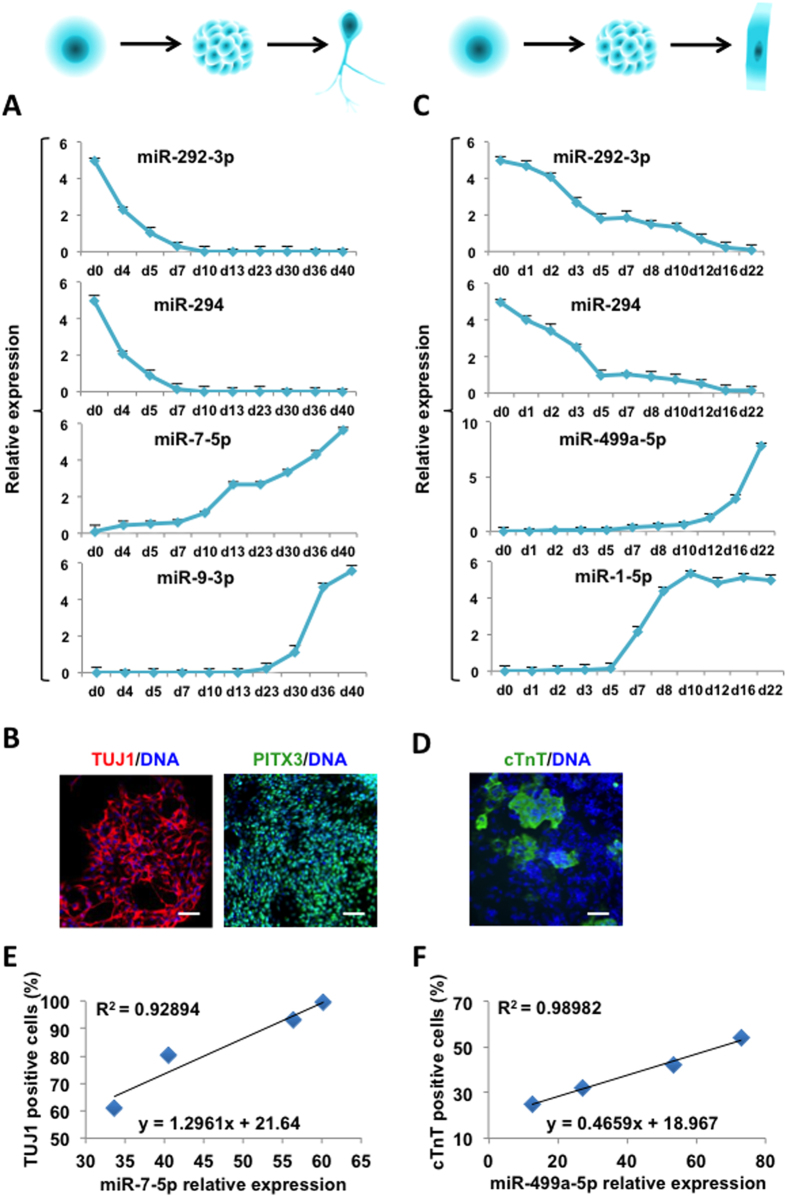
MiRNA expression detection in cell culture mediums along cell differentiation. (**A**) Relative expression levels of miRNA-292-3p, miRNA-294, miRNA-7-5p and miRNA-9-3p in cell culture mediums along the neuronal differentiation process evaluated by real-time PCR. (**B**) Immunostaining of TUJ1 and PITX3 protein in differentiated cells at day 40. Expression of TUJ1 (red) and PITX3 (green) proteins was detected. DNA was stained by Hoechst 33342 (blue). (**C**) Relative expression levels of miRNA-292-3p, miRNA-294, miRNA-499a-5p and miRNA-1-5p in cell culture mediums along the cardiomyocyte differentiation process evaluated by real-time PCR. (**D**) Immunostaining of cTnT protein in differentiated cells at day 22. Positive cTnT (green) was observed, DNA was stained by Hoechst 33342 (blue). (**E**) Correlation analysis of miRNA-7-5p relative expression in cell culture mediums and the percentage of TUJ1 positive cells at day 30, day 35, day 40 and day 45 during neuronal differentiation process. (**F**) Correlation analysis of miRNA-499a-5p relative expression in cell culture mediums and the percentage of cTnT positive cells at day 12, day 15, day 18 and day 21 during cardiomyocyte differentiation process.

To evaluate the feasibility of determining the status of differentiated cells through detecting cell culture medium miRNAs, we differentiated human embryonic stem cells (hESCs) *in vitro* towards the neural and cardiomyocytes (CMs) lineage, and detected the expression of miRNAs in cell culture mediums. The abundance of the ESC highly expressed miRNAs, miR-292-3p and miR-294, decreased along the differentiation process, whereas the abundance of the neuronal specific miRNAs, miR-7-5p and miR-9-3p, increased gradually (Fig. 3A). Similarly, in the cardiomyocytes lineage differentiation process, the abundance of the cardiomyocytes specific miRNAs, miR-1-5p and miR-499a-5p, increased gradually (Fig. 3C). The lineage specifically expressed proteins, TUJ1 and PITX3 in immature and mature neurons as well as Cardiac troponin T (cTnT) in cardiomyocytes, were examined as markers for the success differentiation of the neuronal and cardiomyocyte lineages, respectively (Fig. 3B,D). To demonstrate this method can evaluate the differentiation efficiency of neurons and cardiomyocytes, the percentage of neuronal lineage specific marker TUJ1 was chosed to represent neurons differentiation efficiency as well as the cardiomyocyte lineages specific marker cTnT for cardiomyocytes differentiation efficiency. We detected miR-7-5p and miR-499a-5p relative expression to ESCs in cell culture mediums and the percentage of TUJ1 and cTnT positive cells at four time points during differentiation process, respectively (Fig. 3E,F). We further get the equation between miRNA expression in cell culture mediums and the differentiation efficiency of neurons and cardiomyocytes. Through these equations, we can evaluate the differentiation efficiency of neurons and cardiomyocytes by detecting specific miRNAs expression in cell culture medium (Fig. 3E,F).

## Discussion

The traditional methods to examine cell status all require the destruction of cells. However, given the heterogeneity of cultured cells and the limited resources of certain cells, such destructive methods have several limitations, the most prominent one is inability to achieve ‘*in situ*’ examination, which means that the cells being used for status examination are not feasible for other applications, therefore cells being transplanted into patients or used for other purposes are not guaranteed to have the same status as the examined ones. Here we have demonstrated that the abundance of miRNAs in cell culture medium can be used to examine the type and status of cells, which offers a non-destructive cell examination method and solves the above mentioned problems. We also showed that the relative abundance of miRNAs in cell culture mediums were constant in different cell density and the ratios of the miRNA amount in culture mediums to that in cells were also constant under different cell culture conditions.

The method is feasible for both pluripotent and differentiated cells without species limitations. Although we also detected the presence of some mRNAs from cell culture medium, their abundance is lower than that of miRNAs and the expression correlation between cellular and culture medium mRNAs is not as well as that of miRNAs, probably due to the rapid degradation of mRNAs in culture mediums. We also proved that when appropriate miRNA is chosen, the cell culture medium miRNA detection method is capable to distinguish cells with different levels of pluripotency, therefore offers an efficient way to identify targeted iPSCs. Similarly, the method can also be used to evaluate the status of cells generated by differentiation, trans-differentiation or other cell fate conversion techniques. Taken together, the method developed by this work is capable to determine the type and status of cells with relatively high sensitivity, specificity as well as high efficiency, which is of great value for basic researches and clinical therapies requiring prior determination of cell status.

## Methods

### Cell culture and differentiation

MEFs, TTFs, hFFs, human breast cancer cell line MCF-7, human hepatocellular cell line BEL-7402 and human lung adenocarcinoma A549 cells were grown in DMEM supplemented with 10% FBS. Generation of mouse pluripotent iPS cell lines were performed as described previously^18^. All mouse pluripotent stem cell medium, iPSC induction medium and KOSR MEF cells medium were 400 ml DMEM/F12 supplemented with 100 ml KOSR, 5 ml 200 mM (100×) Glutamine, 5 ml (100×) β-mercaptoethanol, and 50 μl LIF. 2i mouse pluripotent stem cell medium were N2B27 + 2i media with LIF. The 2i treatment included the addition of Mek inhibitor PD0325901 (1 μM) and GSK3 inhibitor CHIR99021 (3 μM). N2B27 medium consisted of DMEM/F12 and neurobasal at a 1:1 ratio, 1% N2 supplement, 1% B27 supplement, 2 mM L-glutamine, 1% nonessential amino acids, 100 U/ml penicillin, 100 mg/ml streptomycin, 0.1 mM β-mercaptoethanol, and 5 mg/ml bovine serum albumin (BSA). For different cell density assay, different numbers (1, 2, 5, 10 × 10^5^) of cells were cultured in 6 cm cell culture dish individually. Human iPSCs were generated from hFF cells transfected with expression constructs for Oct4, Sox2, Klf4 and c-Myc by dox-inducible lentiviral-mediated expression systems as described^19^. All human pluripotent stem cell medium and iPSC induction medium were 400 ml knockout-DMEM supplemented with 100 ml KOSR, 5 ml NEAA (Life Technologies), 5 ml 200 mM (100×) Glutamine, 5 ml (100×) β-mercaptoethanol, and 4 ng/ml bFGF. For neuronal differentiation, hESCs (H9) were differentiated into midbrain dopamine (DA) neurons as described previously^20^ and examined using immunostaining after 40 days. For cardiomyocyte formation, hESCs were differentiated into cardiomyocyte as described previously^21^ and examined using immunostaining on day 22.

### Immunofluorescence and alkaline phosphatase analysis

Antibody stained cells were fixed with 4% paraformaldehyde for 10 min and then permeabilized with 0.5% Triton X-100 for 10 min followed by blocking with 5% donkey serum (Jakson) in PBS. The cells were incubated with primary antibodies against Oct4 (Santa Cruz) overnight at 4 °C, followed by the use of secondary antibodies at room temperature for 1 h. Finally, DNA was stained with hoechst 33342 (Life Technologies) for 5 min. Cells were examined under a confocal microscope (Zeiss, LSM 780 META). Detection of alkaline phosphatase, an indicator of undifferentiated ESCs, was carried out using a BCIP/NBT Alkaline Phosphatase Colour Development Kit (Beyotime) following the manufacturer’s instructions.

### Cell culture medium collection

MEFs, TTFs, hFFs, human cancer cell lines, mouse and human pluripotent stem cells were incubated at 37 ^o^C in a 5% CO_2_ incubator overnight. Supernatant was collected after overnight culture from dish and filtered through a 0.45 μm filter for RNA extraction.

### RNA extraction from cell culture medium

For cell culture medium RNA isolation, equal volume of Trizol was used, and three steps of phenol/chloroform purification were performed due to the high amount of proteins in cell culture medium. In general, 0.5–1 μg RNA could be obtained from 5 ml cell culture medium. Total RNA was used for real-time PCR experiment.

### Real-time PCR detection of mature miRNAs

Total RNA was isolated as described above. Reverse transcription of 10 μl purified cell culture medium RNA into cDNA was carried out using an All-in-One^TM^ miRNA First-Strand cDNA Synthesis Kit (Genecopoeia). Subsequent real-time PCR, using miR-specific primers and universal adaptor PCR primers (Genecopoeia), was performed with a Stratagene Mx3000p QPCR System (Genetimes). The reactions were incubated in a 96 well plate at 95 °C for 10 min, followed by 40 cycles of 95 °C for 15 s and 60 °C for 1 min. All reactions were run in triplicate.

### RNA extraction and real-time PCR

Total RNA was isolated using TRIzol Reagent (Invitrogen, USA) according to the manufacturer’s protocol. Afterwards, 1 ug of RNA from each sample was extracted and reversely transcribed into cDNA using random primers and subjected to real-time PCR experiment. Real-time PCR was performed using a Stratagene Mx3000p QPCR System (Genetimes). The primer pairs used were: Oct4 forward primer, 5′- GCAGGAGCACGAGTGGAAAGCAAC-3′ and reverse primer, 5′- CAAGGCCTCGAAGCGACAGATG-3′; and Gapdh forward primer, 5′-TCCCACTCTTCCACCTTCGATGC-3′ and reverse primer, 5′-GGGTCTGGGATGGAAATTGTGAGG-3′.

### Diploid blastocyst injection

Diploid blastocysts were collected from E3.5 time-pregnant mice. iPS cells were injected into diploid blastocysts that were transferred to CD-1 pseudopregnant recipient females. Then the adult chimeric offsprings were mated to a CD-1 mouse.

### Tetraploid embryo complementation

Tetraploid embryo complementation was carried out by injecting iPS cells (B6D2F1, black coat origin) into CD-1 tetraploid embryos (white coat colour). Embryos derived from tetraploid blastocyst injection (4 N) were dissected in handling media on the day of birth (E19.5).

### Statistical analysis

All real-time PCR experiments were performed in triplicate, and all presented experiments have been repeated for several times. Data shown are presented as means ± SE (standard error) of three or more independent experiments. Student’s t test was used for statistical analyses for experimental results. For correlation analysis, the cell or culture medium miRNA expression values relative to Gapdh in the qPCR assay were transformed to log2 values. The transformed expression values were used for scatter plots and Pearson’s correlation analysis. The correlation coefficients and the corresponding p-values were calculated by the cor.test function of R.

## Additional Information

**How to cite this article**: Zhang, Y. *et al*. A non-invasive method to determine the pluripotent status of stem cells by culture medium microRNA expression detection. *Sci. Rep.* **6**, 22380; doi: 10.1038/srep22380 (2016).

## Supplementary Material

Supplementary Information

## Figures and Tables

**Table 1:** Pluripotency evaluation of ES and iPS cell lines used in the experiments.

Cell line	Nuclear donor	Genetic background	Chimera formation (germline transmission)	4n injection (%)			
				Injected blastocysts	Embryos arrested at E10.5–13.5	Embryos arrested at E15.5–17.5	Live pups
IP20D-3	MEF	B6 × D2 F1	√(√)	166	8(4.8)	0	0
IP36D-3	MEF	B6 × D2 F1	√(×)	143	4(2.8)	0	0
IP14D-1	MEF	B6 × D2 F1	√(√)	144	5(3.5)	0	5(3.5)
IP14D-6	MEF	B6 × D2 F1	√(√)	132	1(0.8)	0	3(2.3)
IP16DT-2A	TTF	B6 × D2 F1	√(√)	110	0	0	4(3.6)
IP14DN-5	NSC	B6 × D2 F1	√(√)	142	0	0	8(5.6)
ESC2	Fertilized egg	B6 × D2 F1	√(√)	120	0	1(0.8)	4(3.3)
CL11	Fertilized egg	B6 × D2 F1	√(√)	92	3(3.3)	0	2(2.2)

√ indicates positive results, and × indicates negative results. TTF, tail tip fibroblasts; NSC, neural stem cells.
