# Supplementary material for: A non-invasive method to determine the pluripotent status of stem cells by culture medium microRNA expression detection
Source: Sci Rep. 2016 Mar 1;6:22380. doi: 10.1038/srep22380 (PMC4772130; doi:10.1038/srep22380)
Supplement: Supplementary Information [file srep22380-s1.pdf]

A non-invasive method to determine the pluripotent status of stem cells by culture medium microRNA expression detection

Ying Zhang, Gui-Hai Feng, Kai Xu, Libin Wang, Peng Cui, Yuhuan Li, Chenxin Wang, Fei Teng, Jie Hao, Hai-Feng Wan, Yuanqing Tan, Xiu-Jie Wang and Qi Zhou

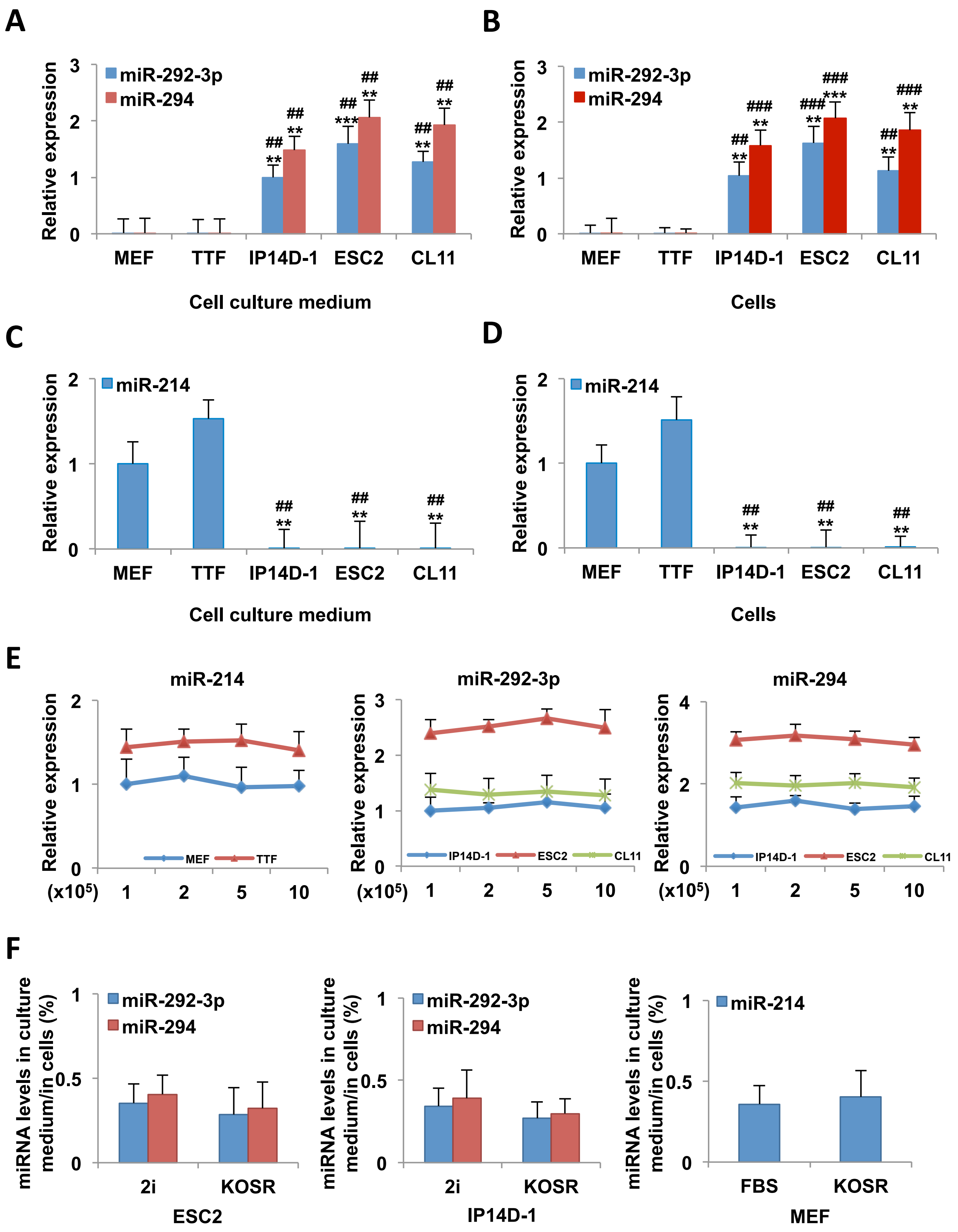
A
Relative expression
miR-292-3p
miR-294
MEF
TTF
IP14D-1
ESC2
CL11
Cell culture medium
B
Relative expression
miR-292-3p
miR-294
MEF
TTF
IP14D-1
ESC2
CL11
Cells
C
Relative expression
miR-214
MEF
TTF
IP14D-1
ESC2
CL11
Cell culture medium
D
Relative expression
miR-214
MEF
TTF
IP14D-1
ESC2
CL11
Cells
E
miR-214
miR-292-3p
miR-294
Relative expression
MEF
TTF
IP14D-1
ESC2
CL11
(x10⁵) 1 2 5 10
F
miRNA levels in culture medium/in cells (%)
miR-292-3p
miR-294
miR-214
2i
KOSR
FBS
ESC2
IP14D-1
MEF

Figure S1. **MiRNA expression abundance in mouse cells and cell culture mediums.** (**A**) Relative expression levels of miRNA-292-3p and miRNA-294 in cell culture mediums. (**B**) Relative expression levels of miRNA-292-3p and miRNA-294 in cells. (**C**) Relative expression levels of miRNA-214 in cell culture mediums. (**D**) Relative expression levels of miRNA-214 in cells. **(E)** Relative expression levels of miRNA-214 (left), miRNA-292-3p (middle) and miRNA-294 (right) in cell culture mediums with different (1, 2, 5, 10×10^5 cells per 6 cm dish) cell density. **(F)** The ratio of the miRNA-292-3p and miRNA-294 amount in culture mediums to that in ESCs (ESC2) under 2i and KOSR culture conditions (left). The ratio of the miRNA-292-3p and miRNA-294 amount in culture mediums to that in iPSCs (IP14D-1) under 2i and KOSR culture conditions (middle). The ratio of the miRNA-214 amount in culture mediums to that in MEFs under FBS and KOSR culture conditions (right). The miRNA expression in all experiments was evaluated by real-time PCR.

** $P < 0.01$, ***$P < 0.001$, compared to MEF, ## $P < 0.01$, ###$P < 0.001$, compared to TTF, the Student's t-test. All the data represent three independent experiments.

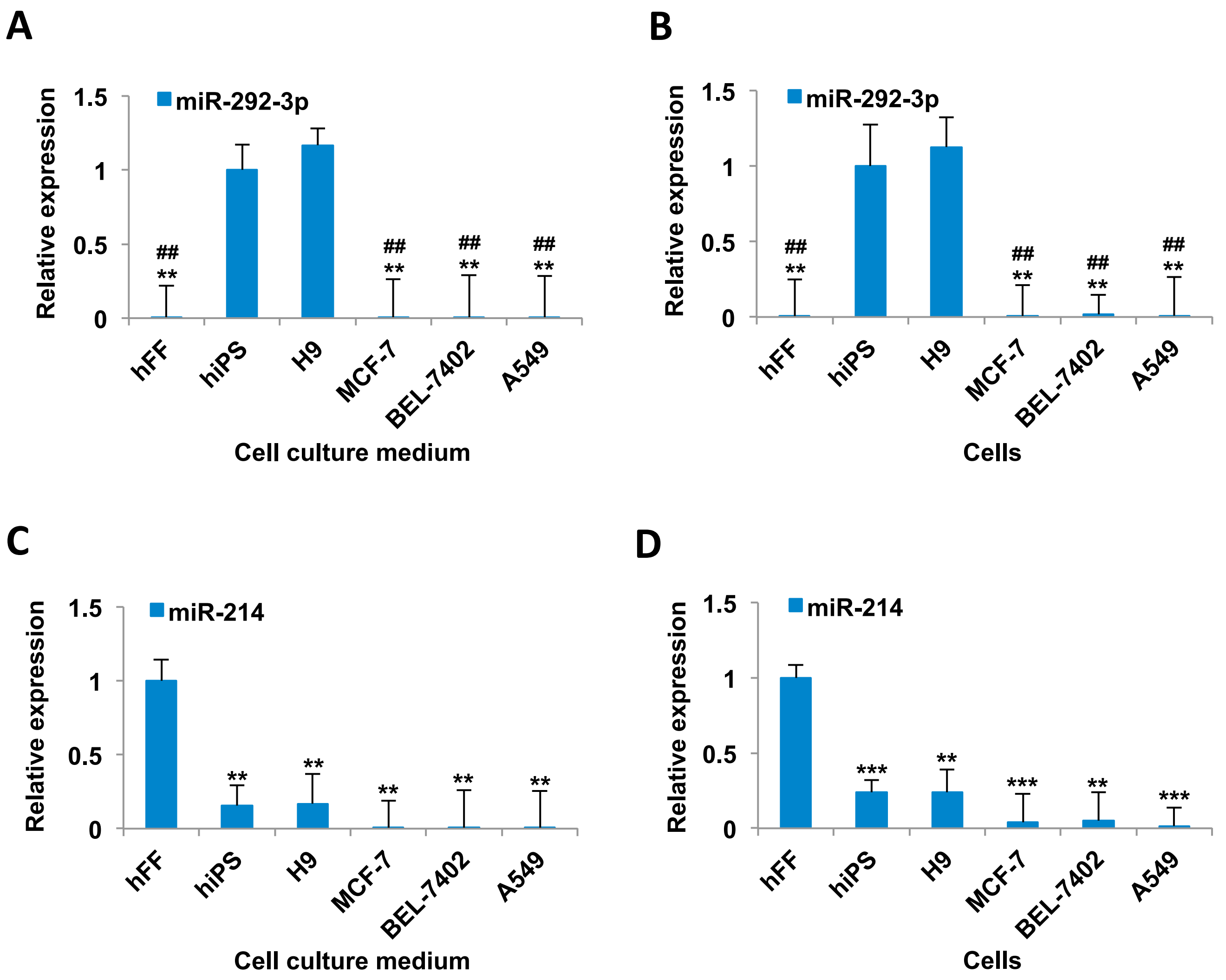
A
B
C
D
miR-292-3p
miR-214
Relative expression
1.5
1
0.5
0
##
**
***
hFF
hiPS
H9
MCF-7
BEL-7402
A549
Cell culture medium
Cells

Figure S2. MiRNA expression detection in human cells and cell culture mediums. (**A**) Relative expression levels of an ESC/iPSC specific miRNA, miRNA-292-3p, in the cell culture mediums. (**B**) Relative expression levels of miRNA-292-3p in cells. (**C**) Relative expression levels of a fibroblast highly expressed miRNA, miRNA-214, in cell culture mediums. (**D**) Relative expression levels of miRNA-214 in cells. The miRNA expression in all experiments was evaluated by real-time PCR.

** $P < 0.01$, ***$P < 0.001$, compared to human iPSC, ## $P < 0.01$, compared to H9, the Student's t-test. All the data represent three independent experiments.
